# Dysphagia Care Processes on Acute Stroke Wards: An Ethnographic Study of Barriers and Facilitators Relevant to Stroke‐Associated Pneumonia

**DOI:** 10.1111/1460-6984.70295

**Published:** 2026-07-25

**Authors:** Sabrina A. Eltringham, Craig J. Smith, Ben Bray, Emma Richards, Jo Burke, Sue Pownall

**Affiliations:** ^1^ Speech and Language Therapy Department Sheffield Teaching Hospitals NHS Foundation Trust Sheffield UK; ^2^ Centre for Applied Health & Social Care Research (CARe) Sheffield Hallam University Sheffield UK; ^3^ Division of Cardiovascular Sciences Manchester Academic Health Science Centre University of Manchester Manchester UK; ^4^ Manchester Centre for Clinical Neurosciences Geoffrey Jefferson Brain Research Centre Salford Royal Hospital Northern Care Alliance NHS Trust Manchester UK; ^5^ Department of Population Health Sciences King's College London London UK; ^6^ Neurosciences Department Sheffield Teaching Hospitals, NHS Foundation Trust Sheffield UK; ^7^ Combined Community and Acute Care Group (CCAG) Research Centre Sheffield Teaching Hospitals NHS Foundation Trust Sheffield UK

**Keywords:** care processes, dysphagia, stroke, stroke‐associated pneumonia

## Abstract

**Introduction:**

Dysphagia is associated with increased risk of stroke‐associated pneumonia (SAP). The multifactorial pathophysiology of SAP and the interplay of care processes make it challenging to unpack which components are associated with risk of SAP. The aim of the research was to capture contextual aspects of dysphagia management to build knowledge of care processes relevant to SAP.

**Methods:**

The methodology was an ethnographic approach. The method was participant observation. Implementation of specialist swallow recommendations, positioning, and oral care processes of 10 stroke patients was observed around mealtimes during the first 72‐h of hospital admission. Data was analysed thematically. People affected by stroke were involved in the data analysis process and in the co‐generation of themes and implications for clinical practice.

**Results:**

Four themes were generated from the data: 1. Patient preparation for mealtimes and medications; 2. Variability in resources, knowledge and implementation of dysphagia care; 3. Swallowing and oral care are everyone's business; and 4. Communication about the person's dysphagia management plan and staff attitudes.

**Conclusions:**

This research enhances understanding of environmental barriers and facilitators that shape how dysphagia care is enacted in acute stroke settings, and how variability in everyday practice may influence circumstances relevant to SAP risk. The findings highlight the role of communication processes, patient and carer information needs, staff awareness of dysphagia diets, mealtime preparation, oral care practices, and access to resources in supporting the implementation of swallowing management plans within routine care.

**WHAT THIS PAPER ADDS:**

*What is already known on the subject*

- The pathophysiology of stroke‐associated pneumonia (SAP) is multifactorial.
- Patients are most susceptible to SAP during their first 72 h post‐stroke.
- Preventative measures to identify dysphagia as early as possible, such as early dysphagia screening and a specialist swallowing assessment, are associated with reduced risk of SAP.

*What this paper adds to the existing knowledge*

- Increased understanding of how dysphagia care processes may impact on SAP.
- Identifies environmental barriers and facilitators to minimising potential risk of SAP, specifically relating to the implementation of the specialist swallowing assessment recommendations, positioning and oral care.
- Identifies areas for clinical improvement.

*What are the potential or clinical implications of this work?*

- Swallowing management plans are complex and often only partially enacted, with key elements beyond diet and fluid modification frequently overlooked.
- Dysphagia management requires a genuinely multidisciplinary and system‐wide approach, involving all ward staff and informed family and carers.
- Effective implementation depends on system‐level factors which include: clear, consistent and up‐to‐date sharing of swallowing plans across all channels; staff, patient and carer education; access to appropriate resources; and supportive ward practices and attitudes.

## 1 Introduction

Stroke‐associated pneumonia (SAP) encompasses a spectrum of lower respiratory tract infections that occurs within the first 7 days of stroke onset (Smith et al. 2015), occurring frequently in around 14% of patients (Kishore et al. 2015). It is associated with increased risk of mortality, prolonged length of hospital stay, and has considerable impact on healthcare resources (Ali et al. 2017). The susceptibility to post‐stroke pneumonia within the first 72 h of stroke has considerable implications for therapeutic intervention. Preventing SAP requires very early intervention strategies after stroke onset to have a pathophysiological and clinical impact, particularly as clinical manifestations of SAP lag behind its pathophysiological evolution (Smith 2021).

Dysphagia is a significant risk factor for SAP, and in patients who aspirate, the risk increases 11‐fold (RR 11.56) (Martino et al. 2005). Preventative measures to identify dysphagia and reduce aspiration, such as early dysphagia screening and a clinical swallowing evaluation (CSE) by a speech and language therapist (SLT), typically conducted in patients who fail the dysphagia screen, are associated with reduced risk (Bray et al. 2017). There is potential for a range of medical interventions and care processes that may influence occurrence of SAP during the acute phase of stroke in patients with dysphagia. There is limited evidence on what measures or interventions may reduce this risk (Eltringham et al. 2020).

This research builds on a programme of research that focused on the links between organisational practices in dysphagia and SAP (Eltringham et al. 2020; Eltringham et al. 2018; Eltringham, Smith, et al. 2019; Eltringham, Pownall, et al. 2019; Eltringham et al. 2022). The aim of this ethnographic study was to explore how dysphagia care processes are enacted in acute stroke care, and to identify contextual barriers and facilitators within everyday practice that may shape conditions relevant to SAP risk. The study also sought to identify practices and system‐level factors that could inform future quality improvement initiatives in acute stroke care. If areas for potential intervention are identified these could be taken forward either as a clinical trial or in a quality improvement method (Jones et al. 2021).

## 2 Methods

### 2.1 Qualitative Approach and Research Paradigm

The methodology was an ethnographic approach, and the method was participant observation (O'Reilly 2009), with the emphasis being on observation on the participant‐observation continuum. Ethnography can be useful in the context of healthcare improvement by encouraging reflection, problem solving and exposing hidden practices (Black et al. 2021). The extent of the researcher's (S.E.) participation was to gain access and to be close enough to be able to collect data by reflexive observation and asking questions as they arose rather than through immersion. The researcher's philosophical approach is shaped by a post‐positivist world view. The ontology of post positivism is that reality exists, but there may be limits in our ability to measure it, and the researcher is building an approximation of the object of research, but never quite an absolutely truthful picture of it.

People affected by stroke were actively involved throughout the research cycle to ensure the relevance, quality and impact of the research were consistent with best practice guidance on Patient and Public Involvement (PPI) in health research (NIHR 2024). The research question aligns with the Stroke Priority Setting Partnership priority of ‘reducing complications post‐stroke’ (www.jla.nihr.ac.uk/priority‐setting‐partnerships/stroke), and public representatives were involved at the commissioning stage. Two Patient and PPI groups, including a panel of stroke survivors with aphasia and their carers, contributed to the study design, development of participant materials, analysis and interpretation, and dissemination of the research findings. This approach reflects growing evidence that involving people with lived experience enhances the credibility, relevance and rigour of findings (Eltringham, Pownall, et al. 2019; Volmer and Broomfield 2022).

PPI contributors made several tangible impacts on the study. For example, they refined the Aphasia Friendly Participant Information Sheet, enhancing clarity of language and improving accessibility through the inclusion of alternative formats to support individuals with visual impairments, thereby increasing inclusivity for stroke survivors. They were actively involved in validating researcher interpretations of the data and generating additional insights, which strengthened the credibility and depth of the analysis. Furthermore, they reviewed dissemination materials to ensure that study findings were communicated in ways that were accessible, relevant, and meaningful to intended audiences. Collectively, these contributions demonstrate how PPI input directly improved study accessibility, enriched data interpretation, and ensured that outputs were both relevant and usable for people affected by stroke.

### 2.2 Researcher Characteristics and Reflexivity

This study is situated within interpretive ethnography, which recognises that knowledge is co‐produced through the researcher's engagement with the field rather than discovered through detached observation (O'Reilly 2009; Emerson et al. 2011). As a practitioner‐researcher, the first author's (S.E.) clinical expertise shaped both what was observed in the field and how interactions were interpreted. Rather than treating subjectivity as bias to be eliminated, it was approached reflexively as a resource for analytic insight.

The researcher (S.E.) is an SLT employed at the hospital where the research took place. Her clinical background and familiarity with the context enabled her to interpret complex care interactions and understand the nuances of practice around mealtimes and dysphagia management. S.E. was aware that her professional role could both enrich and bias the data collection and interpretation. Sensitive to this she maintained a reflexive stance throughout the study, using field notes to explore her insider‐outsider position and to document moments when her assumptions as an SLT were either reinforced or challenged. She actively engaged with counter evidence and reflected on how such moments generated new or revised understandings. Observing through a disciplinary lens was recognised as inevitable but was treated not as a limitation, but as a tool for deeper insight, provided it was critically examined. S.E. took steps to reduce role confusion during observations by wearing a research badge, not her clinical uniform, and ensuring signage made her non‐clinical role explicit, although it is acknowledged that colleagues may have still positioned her by role.

### 2.3 Context

The setting for the observations was a centralised Hyper Acute Stroke Unit (HASU), and Acute Stroke Unit (ASU) in an NHS Trust in England. Observations were prioritised during the first 72 h of admission and took place in the bay or side room where the stroke patient's bed was located from 7 November 2022 to 20 February 2023.

### 2.4 Sampling Strategy

A purposeful sampling method was used. A Stroke Research Nurse (SRN) identified patients consecutively admitted to the HASU based on the study inclusion criteria (Table S1) and used information from the medical records and discussion with the dysphagia trained practitioner working on the unit to clarify the outcome of the CSE. Data from a previous clinical audit (CEU Registration Number 8044) was used to ensure a representative sample of patients was recruited based on the outcome of the CSE adjusted for the study's inclusion/exclusion criteria. The sample size was ten stroke patients. The sample size for the observations was based on the context of the preceding programme of research (Eltringham, Smith et al. 2019; Eltringham, Pownall, et al. 2019; Eltringham et al. 2022) and the rich amount of data generated from the methodology.

### 2.5 Ethical Approval

The research obtained ethical approval from Wales REC4 Research Ethics Commitee (REC reference 22/WA/0246). Potential patient participants were approached and provided with information about the study. Written consent was obtained before participation. Consent to participate was sought from a next of kin via a consultee process in participants who lacked mental capacity. Written informed consent was sought from staff working on the HASU who were assigned to provide routine care to the identified patient participants. A sign alerted visitors and other professionals not directly involved in their care that observations were taking place and if they did not wish to be involved to contact the researcher. An amendment was submitted to the REC to meet the recruitment target. The amendment was to include patients who were not registered with a local General Practitioner. These patients had previously been excluded as it was anticipated they would be transferred to their local ASU before observations could be completed within the first 72 h of hospital admission.

### 2.6 Data Collection Methods and Technologies

Observations included the mealtime, ward staffing board, electronic whiteboard and bedside swallowing notices. The Mealtime Assessment Scale (MAS) (Pizzorni et al. 2020) was used as an observation tool. The MAS draws on the World Health Organisation's ICF framework to evaluate mealtimes. It comprises an initial section to record demographic information and medical history of the patient and four subscales: (1) Structures, functions and activities influencing the meal; (2) Environmental Factors influencing the meal; (3) Swallow safety during the meal and (4) Swallow efficacy during the meal (Table S2). For the purposes of the study the MAS was not used to assess the safety and efficacy of the person's swallow but as a structured observation tool as part of the data collection methods. The mealtime observations were complemented with field notes and information from the participants medical records. Information about the patient's swallowing management plan was extracted from the medical records, bedside swallowing notice and electronic whiteboard. The ward staffing board provided information on staffing levels (actual vs. planned) and staff assigned to each bay/room. Medical records were monitored for possible development of pneumonia and oral care intervention. Field notes included scene setting, a diagram of the room, a commentary of events, specific quotations from the participants and staff delivering the routine care, and S.E.’s reflections.

### 2.7 Units of Study

The units of study included mealtime observations of 10 stroke patients and NHS health professionals who were involved in delivering routine care during mealtimes. Staff included nursing, therapy staff [SLTs, Physiotherapists (PTs) and Occupational Therapists (OTs)] and clinical support staff. Two separate observations for each patient took place at different mealtimes and days of the week during the first 72 h of hospital admission. The timing of the first observation was the first mealtime after a participant had given their informed consent. The care processes identified for further exploration were oral care, implementation of the SLT's swallowing management plan following the CSE and positioning at mealtimes.

### 2.8 Data Processing

The MAS observation template and fieldnotes were completed in paper format in the observation setting. This information and information from the participant's medical records were transferred onto an electronic Case Report Form (eCRF) and an Excel spreadsheet stored in a secure electronic database stored on a secure server approved by Trust Information Governance.

### 2.9 Data Analysis

There were three iterative stages to the analysis, which drew on principles of thematic analysis with an interpretive lens informed by post‐positivism. The aim was not only to categorise observations but to generate meaningful insights into how environmental and care process factors may influence SAP risk.

**Stage 1: Familiarisation and initial coding**

The researcher (S.E.) immersed herself in the dataset (field notes, MAS observations, medical records) by repeatedly reading the data and identifying patterns of behaviour, interactions and contextual features relevant to swallowing care. She used an inductive approach, allowing data segments to be labelled in the researcher's own terms. Codes were initially grouped descriptively (e.g. ‘supportive family presence’) but were then interrogated for deeper meaning and relationships. S.E. actively searched for and recorded examples that contradicted emerging assumptions. For example, staff member S10 was observed providing optimal dysphagia care while managing four acutely unwell patients in a busy bay.

**Stage 2: Iterative theme development using theoretical lenses**

To move beyond surface‐level categorisation, analysis combined inductive thematic development with theoretically informed interpretation. Initial coding was conducted iteratively and inductively, drawing on principles of reflexive thematic analysis to allow patterns to emerge from the data (Braun and Clarke 2006). Subsequently, codes were interpreted using sensitising theoretical frameworks, consistent with qualitative approaches that use theory to extend analytic depth rather than constrain it (Blumer 1954).

Codes were mapped onto the World Health Organisation's ICF framework. Mapping codes onto the ICF was not intended to categorise findings mechanistically, but to support interpretation of how swallowing care is shaped by interactions between a person's health condition, the mealtime activity and their contextual factors (International Classification of Functioning 2001). The researcher used her clinical‐academic experience of the Facial Oral Tract Therapy (FOTT) ‘Swallowing and eating’ and ‘Oral hygiene’ algorithms (Hansen and Jakobsen 2010) to consider the ward environment, including patient location for mealtimes, furniture, objects and aids.

These frameworks served as heuristic devices to frame environmental barriers/facilitators not simply as present or absent, but as embedded in systems of care and professional assumptions. For example, ‘incorrect patient positioning’ was not only noted as a deviation from recommendations but interpreted in the context of resource constraints, staff training and patient autonomy. Tensions and contradictions in the data were intentionally retained to inform theme refinement.

**Stage 3: Co‐analysis with people affected by stroke**

Initial themes were presented to two PPI members, alongside anonymised excerpts from field notes and observation summaries. To facilitate meaningful input, S.E. co‐designed the session format with the group's coordinator (J.B.), using accessible materials and an online discussion board. Group members sense checked interpretations, challenged assumptions (e.g. around independence and dignity at mealtimes), and contributed alternative framings of the data based on lived experience. Their feedback led to the generation of new subthemes (e.g. the emotional impact of isolation during mealtimes in side rooms) and nuanced the implications of the findings.

### 2.10 Theme Consolidation and Naming

Themes were refined to ensure they were conceptually clear and firmly grounded in the data. Each theme was defined, named, and illustrated with data extracts that captured both typical and deviant cases. Attention was given to ensuring that themes represented interpretive insights rather than mere summaries of observed actions. S.E. documented decisions, uncertainties, and how her professional lens as an SLT influenced the interpretation.

### 2.11 Techniques to Enhance Trustworthiness

Several techniques were used to ensure trustworthiness. S.E. focused on understanding participants’ perspectives and experiences and wrote her fieldnotes contemporaneously to preserve their distinctive features to avoid homogeneity and flatness that comes from retrospective recall. The members of the PPI group and the research team provided peer validation of the themes. The Standards for Reporting Qualitative Research (SRQR) were used for transparency of reporting (O'Brien et al. 2014). The Guidance for Reporting Involvement of Patients and the Public (GRIPP2) in research was used for the reporting of PPI in the research (Staniszewska et al. 2017).

## 3 Results

Total observation hours were 33:17 (hours: minutes). Observations took place at mealtimes across the day: breakfast (*N* = 7), lunch (*N* = 4) and supper (*N* = 9). Two observations occurred over the weekend. The duration of the observations ranged from 0:45 to 4:15 (hours: minutes). The average duration of the meal from when the patient took their first bite to when they swallowed their last bite was 18 min.

### 3.1 Participant Characteristics

Ten patients were recruited (1 male and 9 female). All had ischemic strokes. The mean age was 75.64 years, and the median National Institutes of Health Stroke Scale (NIHSS) score was 10. All participants were observed across two mealtimes. The average time from admission to HASU and the 1st mealtime observation was 42:04 (hours: minutes) and 54:00 (hours) for the 2nd observation. The recommended level of assistance required ranged from full assistance to helping as needed. One participant was fully dependent for mealtimes. One participant was newly prescribed antibiotics for chest symptoms, which were discontinued within 7 days. One participant was recommended restricted swallowing trials and referred for urgent alternative feeding. A nasogastric tube was not inserted (see Table 1).

**TABLE 1:** Patient participant characteristics.

ID	P1	P2	P3	P4	P5	P6	P7	P8	P9	P10
Age (Y:M)	73:3	77:0	68:2	72:10	69:8	76:7	79:4	89:4	82:4	67:0
Male:Female	F	F	M	F	F	F	F	F	F	F
Stroke type	LMCA	LMCA	RMCA	LMCA	LMCA	RMCA	RACA	LMCA	RLPT	RMCA
NIHSS	19	3	12	24	23	4	8	15	4	2
Hr:Min adm to 1st observation	58:12	19:56	19:11	43:23	51:53	15:16	37:13	74:32	46:40	51:17
Hr:Min adm to 2nd observation	77:33	23:09	38:13	48:07	66:48	30:44	41:53	89:37	62:08	66:37
IDDSI	L2/L4*	L1/L6	L1/L7	L0/L5	L1/L5	L1/L6	L0/L6	L0/L6	L2/L4	L0/L5
TOMs #	2	3	Missing data	2.5	3	3	3	4	Missing data	3.5
Level of dependency ∼	2	0	1	3	2	2	2	1	1	0

*Notes*: *Swallowing trials, #TOMS Dysphagia Impairment Score (0–5) 0 = Aphagia/5 = No evidence of dysphagia, ∼ MAS Independence in eating score 1st observation (0–3) 0 = completely independent for the whole meal/3 = the patient needs to be spoon‐fed by the carer giver.

Abbreviations: ACAs, anterior cerebral artery; ID, identification; IDDSI, International Dysphagia Diet Standardisation Initiative; L, left; LPT, lenticulostriate perforator territory; MCA, middle cerebral artery; M, month; P, participant; R, right; TOMs, therapy outcome measures; Y, year.

Staff observed providing routine care to the participants during the mealtime observations were Nurses (*N* = 5), Clinical support staff (*N* = 5), SLTs (*N* = 2), PTs (*N* = 2) and 1 Therapy Assistant.

## 4 Themes

Four meta themes were generated: (1) Patient preparation for mealtimes and medications, (2) Variability in resources, knowledge, and implementation of dysphagia care, (3) Eating, drinking, swallowing and oral care is everyone's business and (4) Communication about the person's dysphagia management plan and staff attitudes.

**(1) Patient preparation for mealtimes and medications**

This theme refers to preparing patients for mealtimes and administering medications. This includes giving patients the opportunity to prepare for the meal, such as washing their hands or using hand wipes, sitting patients upright in bed or sitting out in a chair, ensuring that the necessary equipment (e.g. a table, chair and utensils) are in easy reach and providing the recommended level of support and assistance.

Three patients (P6, P7, P8) had specific recommendations about needing assistance to be ‘set up’ for mealtimes. There were examples where patients did not receive the level of support required. One family member commented that, although meals were delivered, support was still needed to set the patient up beforehand. Participant 9 described feelings of isolation while being cared for in a side room. Unable to reposition herself in bed or summon assistance, she expressed discomfort ‘*Everything feels so tender, really sore*’ and she felt thirsty. She did not have a table to place the thickened drink she required, and essential items such as her toothbrush, toothpaste, and water were placed on the cabinet—out of her reach. Reflecting on a moment when oral care was attempted, she recalled: ‘*The one time they did bring my toothbrush and toothpaste, they didn't bring me water or anything to spit out*’ (P9). This account highlights how seemingly minor omissions in care delivery can compound a patient's vulnerability and discomfort, particularly when environmental and functional barriers are not proactively addressed. Other barriers included instances of suboptimal positioning, which resulted in one patient (P6) asking, ‘*Sit me up more*’, poor attention to hand hygiene, and the lack of provision of assistive equipment to facilitate independence around mealtimes for P7, who used adaptive cutlery at home following a previous stroke.

Conversely, there was an example where a PT (S6) integrated mealtime preparation as part of their neurological assessment. They optimised the patient's position ‘*Shall we get you back in your chair*’, checked that the patient could see their food, identified the patient's dislike of the food options being offered, encouraged independence in eating and checked the patient's bedside swallowing notices when the participant's daughter asked if P7 could have bread. Following their assessment, the PT instigated a referral to ophthalmology for suspected visual neglect and said, ‘*I'll mention to SLT that you're not liking the meal*’.

There were also examples of proactive practice during medication administration. Prior to giving P6 her evening meal, one staff member S15, reminded her, ‘*We need to sit you up*’ highlighting awareness of the importance of posture. In another case, P8 received her morning medication from a Staff Nurse (SN) accompanied by a Student Nurse. The SN was observed to consult the handover notes, introduce herself, and clearly explain each medication being administered. She repositioned the bed to sit the patient upright at a 90‐degree angle and delivered the tablets one at a time, with small sips of water—a process that appeared measured and patient‐centred.

By contrast, medication administration for P6 during an early morning round was less carefully managed. The bed was partially adjusted to a more upright position ‘Can I just sit up a bit’ but P6 was visibly drowsy when given her tablets—two at once followed by a larger one—which led to visible difficulty, including coughing and choking. Only after this was, she repositioned to sit more upright. During the observation, she appeared significantly more fatigued compared to the evening meal the day before, commenting, ‘*My muscles feel so weak*’. This episode underscores the vulnerability of patients in acute stroke secondary to reduced alertness, variability in practice and the potential risks when careful attention to swallowing safety is not maintained.

**(2) Variability in resources, knowledge, and implementation of dysphagia care**

This theme captures how variability in staffing, resources, and staff knowledge led to inconsistent implementation of dysphagia and oral care recommendations, with implications for patient safety, dignity and autonomy. The theme is divided into three sub‐themes: (i) Staff knowledge of the International Dysphagia Diet Standardisation Initiative (IDDSI) and implementation of the dysphagia recommendations (ii) Implementation of oral care and (iii) Variation in resources to support the recommended delivery of the swallowing plan and oral care.

(i) *Staff knowledge of IDDSI and implementation of the dysphagia recommendations*

For participants who were recommended IDDSI Level 5 (P4, P5, P10) and Level 6 diet consistencies (P2, P6, P7, P8), there was variation in staff understanding that the Level 5 meal needed to be mashed down to 4 mm pieces, and that the Level 6 meal needed to be cut up into 1.5 cm pieces. There was an instance when bread was served to P7, who was recommended a Level 6 diet. Some staff demonstrated their knowledge of IDDSI Level 5 and explained to the patient that the meal may look different because it's ‘mashed down’. Similarly, there were examples where IDDSI Level 6 meals were cut up and when they were not. There was a lack of awareness by some staff of available Level 5 and Level 6 meal options available from the service meals trolley in addition to the dysphagia diet menus.

In participants who were recommended to have thickened fluids, there were examples where drinks were prepared to the recommended consistency. In other cases, staff were observed to offer drinks that were the incorrect consistency, suggesting a lack of understanding or awareness of flow testing the drink. One participant (P9) was offered a drink which appeared not to have been thickened to recommended Level 2 consistency. P9 coughed saying, ‘*Is that what they call going down the wrong hole?*’ indicating that she may have aspirated some of the drink.

There was variation in staff skill at making drinks with drinks being served with visible lumps of thickener. A barrier to accurately thickening drinks to the required level was the wide variation in cups being used, where there was no ability to accurately measure the quantity of fluids. There was demonstration of safety awareness where the tub of thickener was not left at the patient's bedside for a participant requiring supervision during mealtimes.

(ii) *Implementation of oral care*

Mouth care was delivered by a range of professional groups (PT, OT and Nursing), and relatives also played an advocacy role in prompting care. For example, one family member supported P2 by prompting staff to take her toothbrush and toothpaste to the bathroom. A variety of mouth care practices were observed across participants, including cleaning of the oral cavity and tongue using chlorhexidine‐soaked swabs, removal of secretions, toothbrushing with toothpaste, and application of moisturising gel. However, the consistency of these practices varied, including denture care.

P10 stood out for her proactive approach. She linked her oral hygiene practices to a previous medical experience, explaining that she had a tooth removed prior to a heart valve procedure due to infection risk. This appeared to shape her strong adherence to denture care, including soaking dentures overnight and cleaning them after each meal. In contrast, P8 reported keeping her dentures in overnight and removing them only ‘*sometimes*’ for cleaning, while P7 declined to remove her dentures at all. As a practitioner‐researcher, this observation challenged S.E.’s assumptions that patients would prioritise oral care given its clinical importance and role in overall wellbeing, drawing attention to the complex interplay between clinical guidelines, staff routines and individual agency.

(iii) *Variation in resources to support the recommended delivery of the swallowing plan and oral care*

**FIGURE 1:**
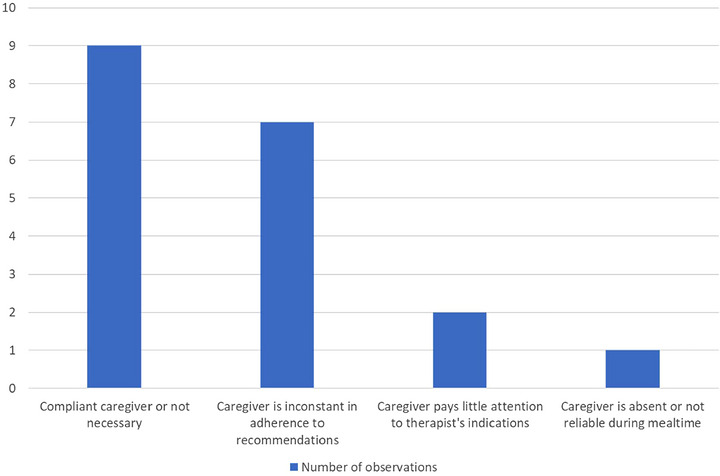
Adherence to the swallowing management plan. Based on MAS item ‘Possibility to rely on caregiver’. The item assesses the presence and compliance of a caregiver to potential alimentary instructions, in case a caregiver is necessary to provide assistance to the patient during the meal. Caregivers may include relatives or nurses. ​Excludes 1 x P2 observation where curtains were drawn around patient's bedside during SLT lunchtime review.

There were 11 different components of the swallowing management plan across the 10 participants. This included the IDDSI diet and fluid consistencies, consideration for urgent alternative feeding, positioning, frequency of swallowing trials (P1), pacing, checking for post‐swallow oral residue, swallow strategies, bolus size, type of equipment/utensils, level of supervision and level of assistance. Mouthcare was not included. There was variation in the adherence to the management plan by the caregiver measured by the MAS component ‘Environmental factors influencing the meal ‘Possibility to rely on caregiver’ (Barrier) (see Figure 1). Accounting for the variances in the provision of the IDDSI levels described in (i) and (ii) there were variations in following advice for participants who were recommended to eat and drink at a ‘slow pace’ (P1, P4, P10), who required supervision (P2, P3, P6, P9, P10) and for those that needed prompting to check or for staff to check for any food left in their mouths (P2, P3, P6, P7, P10). For example, P3 was recommended to have supervision and to check his mouth for any oral residue after meals. His wife reported that he had spat out a lump of battered fish in the sink when he gargled.

There were staffing pressures during the evening mealtime service and at weekends. Family members were observed to assist with setting up meals, one participant's relative commented that the ward appeared short staffed. Relatives were also observed to reaffirm the IDDSI swallowing recommendations and the recommended cup to use. However, there was an example when a visitor of a participant, was observed to try to offer food and drink when the patient appeared drowsy and was turning her head away. In addition to staffing pressures, there was an absence of physical resources such as teaspoons to deliver the recommended bolus size (P4, P8), with one relative commenting that they had to bring in their own spoon, and the use of adaptive cutlery.

The frequency of oral care documented in the patient care plan varied from 1 to 2 times daily to nothing being documented. Mouthcare was not always completed as part of washing and dressing therapy assessments.

**(3) Eating, drinking, swallowing and oral care is everyone's business**

This theme describes the interplay of different stroke professionals and family members in the participants swallowing and oral care, individual professions utilising their specialist knowledge and skills within the stroke multidisciplinary team, and the potential carryover of the person's swallowing management advice into different spaces.

Participants were taken off the ward by therapy staff for kitchen assessments and to the therapy gym, and transferred between wards. Potential barriers included when the patients' swallowing advice was not transferred between settings. For P3, both observations took place on the ASU as they were transferred from HASU within 24 h of their hospital admission. The electronic whiteboard was not updated to reflect that a swallowing assessment had happened on HASU, and that IDDSI recommendations were in place. There was also an example where the medical notes revealed a participant was given a drink during a kitchen assessment which did not comply with their Level 1 recommendations which resulted in an incident being reported on the hospital online system.

Within the stroke interdisciplinary team, staff demonstrated their individual specialist knowledge. For example, therapy staff provided advice in their medical notes to support oral care in a participant (P5) who had limb weakness, incoordination and apraxia: ‘*When brushing own teeth and dentures prompt to ensure brushing teeth with bristles and not back of toothbrush*’ *(S4)*. Other examples included PTs and nursing staff positioning patients to minimise risk of respiratory compromise and an SLT conducting a lunchtime assessment and communicating to P2 and their family about their recommendations before they were discharged home the same day. There were examples where therapy staff adapted their activities to include washing and dressing practice (P1). The medical notes revealed that there were potential missed opportunities for oral care as part of washing and dressing assessments (P7, P8, P9).

Family members were observed to play an active and supportive role in mealtime care, often taking the initiative to implement swallowing recommendations and facilitate safe eating behaviours. In the case of P3, his wife demonstrated a clear awareness of both physical positioning and pacing—key elements of the swallowing management plan. When he was still in bed, she prompted him about eating at the table. She facilitated the transfer by repositioning the table and helping him into the chair. Throughout the meal, she encouraged his independence by placing the fork in his left hand and prompting him to eat. She also actively coached safe swallowing behaviours, guiding him to pace himself and cut smaller pieces, and intervening when he picked up a large piece of fish. Before allowing him to return to bed, she reminded him to let his food go down, demonstrating her awareness of the importance of post‐meal positioning. This example illustrates how family caregivers can become effective partners in dysphagia management when adequately informed and engaged. It also highlights how shared understanding, encouragement, and gentle prompting can support the patient's autonomy while aligning with clinical goals.

**(4) Communication about the person's dysphagia management plan and staff attitudes**

This theme highlights how dysphagia management relied on complex, multi‐layered communication systems and everyday communications between staff, patients and families. Observations demonstrated both supportive practices where staff used communication as an opportunity for education, peer support, and person‐centred care, and potential barriers, where attitudes towards thickened fluids, assumptions about patient preferences, and workarounds around equipment risked undermining swallowing recommendations and patient experience.

There were multiple channels for communicating information about the participants dysphagia management plan, which required updating across the different formats as a patient's recommendations changed. Methods for communication included the IDDSI bedside notices behind the patient's bed, the electronic whiteboard, the ward kitchen noticeboard, the printed nursing handover, face‐to‐face discussions between staff and at ward meetings. One PPI member commented: ‘*I was struck by how much information there is—It needs to be shared simply and easily with so many different people between the different kinds of staff, between the patient and the visitors, and how it needs to be updated when things change and there's the difficulty of doing that with so many different staff…and it's not just nursing staff, there's the kitchen staff and physios*.’

There were positive examples of nursing staff explaining care processes, using their clinical expertise and providing peer support and creating opportunities for education. For example, a nurse (S10) explained to a student nurse how they would make a thickened drink ‘*as and when she [the patient] wants them*’ ‘*is nicer for them*’ as ‘*drinks can get thickened over time*’. They encouraged the student to try thickened drinks for themselves so that they could experience them, talked about the sensation of residue left in the mouth, and used this as an opportunity to provide education about regular mouthcare. Staff were also observed to source alternative preferred food options and were observed to facilitate independence, for example, by holding the yoghurt pot so that a participant (P1) with hemiplegia could feed themselves. Kitchen assistants and clinical support workers discussed suitable modified diet options from the main service trolley, and peer education was provided about suitable IDDSI Level 4 breakfast options between a housekeeper and kitchen assistant for P4.

There were potential attitudinal barriers relating to thickened drinks and spouted beakers. Staff were observed to tell patients ‘*It will taste different you know because it's got thickener in it*’. This had the potential to influence participants attitudes to the drink: ‘*I'm drinking wallpaper paste as it was described last night*’; ‘*I have to have that stuff in it and I don't like it*’; ‘*Not very nice with that thickener*’; ‘*It's like wallpaper paste*’ (P3). There were also examples of potential lack of availability of drinks resulting in participants (P3, P9) stating how thirsty they were. P3's wife understood why thickener could not be left in patient's reach but emphasised that drinks needed to be available. Some staff perceived having a cup with a spouted beaker was easier for the patients and would serve drinks with a beaker lid despite the swallowing advice recommending drinks from an open cup.

## 5 Discussion

This ethnographic study explored how dysphagia care practices were enacted during the acute phase of stroke care, with particular attention around mealtimes. Rather than examining causal relationships with SAP, the study provides insight into the contextual conditions under which dysphagia care is delivered, and how variability in practice, communication, and resources may shape circumstances relevant to SAP risk. Interpretation of the findings was informed by the International Classification of Functioning, Disability and Health (ICF), which supported attention to the dynamic interplay between body functions, activities, participation, and environmental factors during mealtimes. For example, swallowing, conceptualised as a body function, was closely intertwined with mealtime activities and participation, while fluctuations in functions such as fatigue or alertness influenced patients’ ability to sit out for meals or safely engage in the morning medication round. These interactions were further shaped by caregiver actions and ward routines, illustrating how dysphagia care is enacted within a complex, interdependent system rather than through isolated clinical tasks. By focusing on mealtime interactions and care processes as they unfolded in routine clinical work, the findings illuminate how swallowing recommendations are interpreted, prioritised, and implemented within acute stroke care.

A key finding of this study was the variability in how patients were prepared for mealtimes and supported with oral care, particularly among those who needed help ‘setting up’ for mealtimes. Observations showed that when preparation was incomplete, such as inadequate positioning, lack of equipment within reach, or missed opportunities for oral care, patients experienced discomfort, reduced independence and increased reliance on others. From an ICF perspective, these breakdowns illustrate how limitations in environmental support and caregiver actions can constrain participation in mealtime activities. Conversely, when preparation was integrated into routine care by staff across disciplines, patients were better supported to participate safely and independently in mealtimes. These findings extend the work of Jones et al. (2026), who highlighted key challenges experienced by stroke survivors with eating and drinking difficulties in acute stroke units, such as social isolation, insufficient assistance and limited availability of adaptive equipment.

While previous research has identified dependency for feeding and oral care as risk factors for aspiration pneumonia (Langmore et al. 1998; Hibberd et al. 2013), these findings extend that literature by showing how such dependency is created and mitigated through everyday care practices. Preparation for mealtimes functioned as a distributed activity shaped by staffing levels, professional roles, and ward routines rather than as a discrete clinical task. Ensuring access to appropriate equipment and providing therapeutic positioning, supports patients to meet their nutritional, hydration and oral health needs as well as their psychological wellbeing by enabling participation in everyday activities. Facilitating independence in these patients also has the potential to release nursing staff to attend to those patients who are fully dependent and who require 1:1 assistance and to complete other tasks. Integrating oral care and mealtime preparation as part of SLT, PT and OT assessments, such as swallowing, washing, dressing and physical assessments, are practical ways for the multidisciplinary team to address these interlinked domains: functions, activity and participation, within routine care.

The combination of post‐stroke immunosuppression, aspiration of oropharyngeal secretions and stomach contents into the lungs related to impaired consciousness and dysphagia predisposes patients to SAP in the first few days after stroke onset (Hannawi et al. 2013). Observing patients in the hyperacute stroke setting highlighted the susceptibility of patients. Participant's alertness was observed to fluctuate during and between observations, with participants exclaiming how weak and tired they feel. From an ICF perspective, changes in body function such as alertness or low blood pressure interacted with environmental factors and caregiver actions to influence participation in routine activities, rather than determining risk in isolation. The research also highlighted the importance of adhering to all the swallowing recommendations in the patient's management plan, such as the utensils to be used, pacing, checking for oral residue and providing the recommended level of assistance and supervision.

Within this context, the findings highlight how communication practices played a key role in mediating the implementation of swallowing recommendations. This research extends existing literature (Eltringham, Pownall, et al. 2019) by highlighting the need for clear, consistent, and inclusive communication of swallowing plans for patients, families and visitors about managing swallowing problems after stroke, including how the IDDSI levels may change and orientation as to what to look out for on the bedside swallowing notices. Patients and families would benefit from information tailored to their needs and abilities, including visual, aphasia‐friendly, and culturally sensitive formats. The research also highlighted the potential for communication breakdowns between staff and wards—the latter being exacerbated by patients potentially rapidly moving through the pathway. There were multiple modes of communication that were used but were not always aligned, risking delays or errors in implementation. From an ICF‐informed perspective, these communication breakdowns functioned as environmental barriers that shaped how swallowing recommendations were interpreted and enacted in practice.

This study adds to a small body of SLT‐led research that uses an ethnographic approach to explore the everyday realities of clinical care and interpersonal dynamics in hyperacute and acute stroke care (Barnard et al. 2021, 2022). In a study of SLT‐nurse interactions Barnard et al. (2021) found there were ‘small windows in time’ which influenced information sharing and that information sharing practices could be improved by considering how the relevance of information for patient care could be made clearer. The challenges of sharing information for managing swallow safety and keeping swallowing information up to date within ward realities (Barnard et al. 2022) aligns with the observed variability in communication, staff knowledge of IDDSI, and implementation of mouthcare recommendations in our study. By drawing on theory, comparative literature, and experiential knowledge, this study offers a more nuanced understanding of the barriers and facilitators to implementing dysphagia care recommendations. It highlights the need for integrated, team‐based approaches that consider not just clinical protocols, but the organisational and relational factors that shape practice on the ground.

Public and Patient Involvement (PPI) made a substantive and positive influence on the design, conduct and interpretation of this study. People affected by stroke were involved from inception through to dissemination, sense‐checking interpretations, and identifying implications for clinical practice. Their involvement enhanced the credibility and relevance of findings by introducing lived experience perspectives that may not have emerged through professional analysis alone. For example, PPI members highlighted the complexities of communicating dysphagia plans across a range of staff and settings, underscoring systemic challenges not fully captured through observation. A key strength of this approach was the iteration of the researcher interpretations and the generation of new insights by PPI contributors. These contributions underscore the value of embedding PPI throughout research to ensure outputs remain grounded in real‐world experiences.

### 5.1 Study Limitations

The authors acknowledge the potential limitations of the study. The observations of dysphagia care processes took place in a UK single regional hyperacute stroke unit, which may limit applicability to other healthcare systems. However, as an ethnographic study, the findings are not intended to be generalisable but offer transferable insights into how dysphagia care is organised in similar acute stroke contexts. The sample size is small with a limited range of stroke subtypes. The primary author is an SLT, which may have blurred the insider‐outsider boundaries of being a clinician and a researcher (Serrant‐Green 2002). Sensitive to this field, notes were used to mitigate potential bias. There were occasions when the researcher felt the need to intervene for reasons of patient safety or when a member of staff sought their professional opinion. When the latter occurred, the researcher requested the participant defer their question to the SLT on duty. There was the potential for an observer effect, whereby staff behaviour may have been influenced by the presence of the researcher during mealtime observations. Potential for selection bias was minimised by recruiting consecutively admitted patients who met the inclusion criteria and including patients who lacked capacity, and providing accessible participant information sheets to enable patients with communication difficulties to take part. However, patients who are reliant on consultees may influence patients who are included.

While PPI strengthened the study, several limitations warrant consideration. Engagement was primarily with two individuals during data interpretation, which limited diversity in perspectives (e.g. age, stroke severity, cultural background). Broader representation could have offered richer insights and reduced potential bias. Additionally, meaningful involvement required additional resources and coordination, highlighting the need for early planning and flexibility. Future studies would benefit from proactively recruiting a wider range of voices, building in time for training and use of virtual discussion boards.

### 5.2 Conclusions and Implications for Clinical Practice

This ethnographic study explored how dysphagia care practices were organised and enacted in acute stroke care, with a focus on mealtimes. Observations revealed considerable variability in how swallowing recommendations were implemented, shaped by patients’ fluctuating physical capacity and alertness, caregiver availability, communication practices and access to resources.

The findings show that dysphagia care was delivered through interdependent routines involving multiple professional groups and family members, rather than as isolated clinical tasks. Preparation for mealtimes, oral care, and medication administration emerged as distributed activities in which positioning, access to equipment, and timely communication influenced patients’ comfort, independence and participation in everyday activities.

While not designed to establish causal relationships with SAP, the study provides insight into the contextual conditions under which dysphagia care is prioritised and enacted, and how variability in routine practice may shape circumstances relevant to SAP risk.

### 5.3 Clinical Implications

For clinical practice, these findings highlight the need to strengthen communication of swallowing management plans to support shared understanding across teams, alongside ongoing staff education to promote consistent implementation of dysphagia care and oral care. Prioritising effective mealtime preparation, maximising therapy input, and improving patient and carer education are also essential, supported by reliable access to appropriate resources within busy acute stroke settings.

## Funding

Sheffield Hospitals Charity funded this research through a small grant award (Reference no. 202112).

## Conflicts of Interest

The authors declare no conflicts of interest.

## Supporting information




**Supporting Table 1**: Inclusion and Exclusion criteria

## Data Availability

All data supporting the findings of this research are available within the paper and its Supplementary Information.
