# Supplementary material for: Dysphagia Care Processes on Acute Stroke Wards: An Ethnographic Study of Barriers and Facilitators Relevant to Stroke‐Associated Pneumonia
Source: Int J Lang Commun Disord. 2026 Jul 25;61(5):e70295. doi: 10.1111/1460-6984.70295 (PMC13401222; doi:10.1111/1460-6984.70295)
Supplement: Supplementary file 1 — Supporting Table 1: Inclusion and Exclusion criteria [file JLCD-61-0-s001.docx]

**Supplementary Material**

**Table 1: Inclusion and Exclusion criteria**

*Inclusion criteria*

Patients with acute stroke (ischaemic or haemorrhagic) who:

- Live in [Name of city] and are registered with a [Name of city] GP.
- Directly admitted by ambulance to a stroke unit bed on the Hyper/Acute Stroke Unit.
- Have either had a specialist swallowing assessment by a Speech and Language Therapist (SLT) having been identified at risk of dysphagia by a trained nurse using the STHFT Dysphagia Screening Protocol (DSP) OR a specialist swallowing assessment by a Dysphagia Trained Stroke Nurse trained to the equivalent Specialist Level as the SLT on the Inter Professional Dysphagia Framework and have specialist swallowing and have swallowing recommendations in place within 24 hours of admission to hospital. Swallowing recommendations include but are not limited to modification of the consistency of diet and/or fluids as denoted by the International Dysphagia Diet Standardisation Initiative (IDDSI) levels; restricted swallowing trials; patient specific positioning advice, environmental strategies; swallowing manoeuvres and/or strategies; swallowing exercises, oral care/interventions, medication formats/routes.
- Can understand English sufficiently to provide informed consent.
- Are over 18 years of age.
- Consents to enter the study or consultee of patient either a family member or the medical team who gives consents for the patient to participate on their behalf.

*Exclusion criteria*

- Patients not living in [Name of city] and who are not registered with a [Name of city] GP.
- Patients who have onset of stroke symptoms on an outlying ward and not directly admitted to a stroke bed on the Hyper/Acute Stroke Unit.
- Stroke patients who have not had a specialist swallowing assessment by a SLT or Dysphagia Trained Stroke Nurse.
- Stroke patients who have had an assessment by a SLT or Dysphagia Trained Stroke Nurse and are recommended Level 0 Thin Fluids and Level 7 Regular Diet and require no management or who are on their usual fluid and diet recommendations. For example, some older patients or patients with reduced dentition may usually eat a ‘soft and bite sized’ or ‘easy to chew diet,’ or patients with pre-existing swallowing problems may be recommended modified diets and/or fluids by a SLT before their current admission for a stroke and after the specialist swallowing assessment after their stroke they are recommended their preadmission IDDSI diet and fluid recommendations.
- Stroke patients who are recommended 100% Nil by mouth and who are not having oral intake/cautious oral trials only.
- Stroke patients who are palliative or for end-of-life care.

For the purposes of the inclusion/exclusion criteria ‘restricted swallowing trials’ were defined as trials that were structured oral intake and delivered in the context of routine care i.e. mealtimes, with a defined management plan. This was distinct from ‘cautious oral trials’ aimed at assessing readiness for oral intake, not for nutrition/hydration, i.e. tsps/sips only, that were not embedded into mealtimes.

**Table 2 – MAS subscales 1 and 2**

**
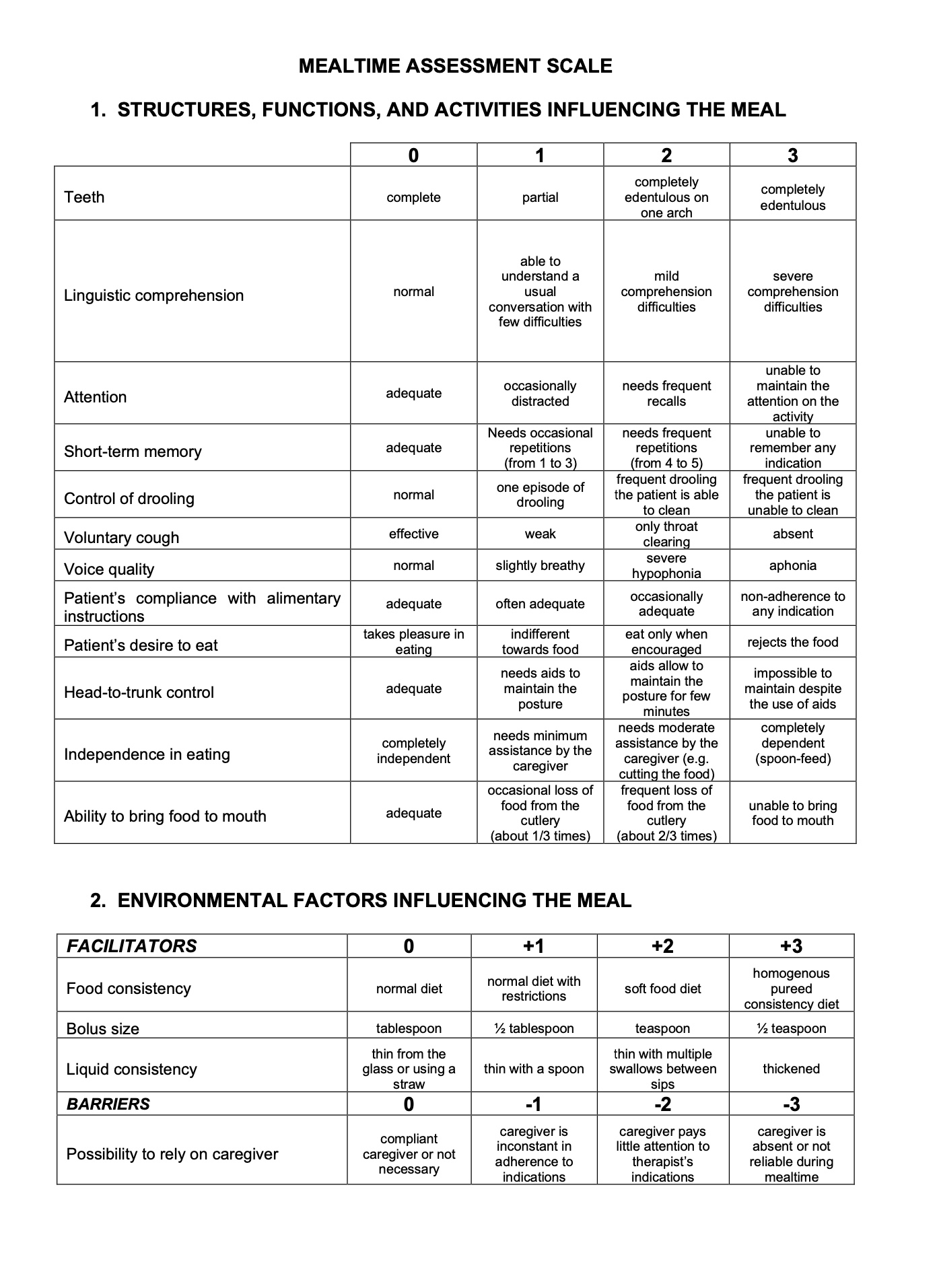
**
